# Supplementary material for: The mechanism of NDM-1-catalyzed carbapenem hydrolysis is distinct from that of penicillin or cephalosporin hydrolysis
Source: Nat Commun. 2017 Dec 21;8:2242. doi: 10.1038/s41467-017-02339-w (PMC5740130; doi:10.1038/s41467-017-02339-w)
Supplement: Supplementary file 1 — Supplementary Information [file 41467_2017_2339_MOESM1_ESM.pdf]

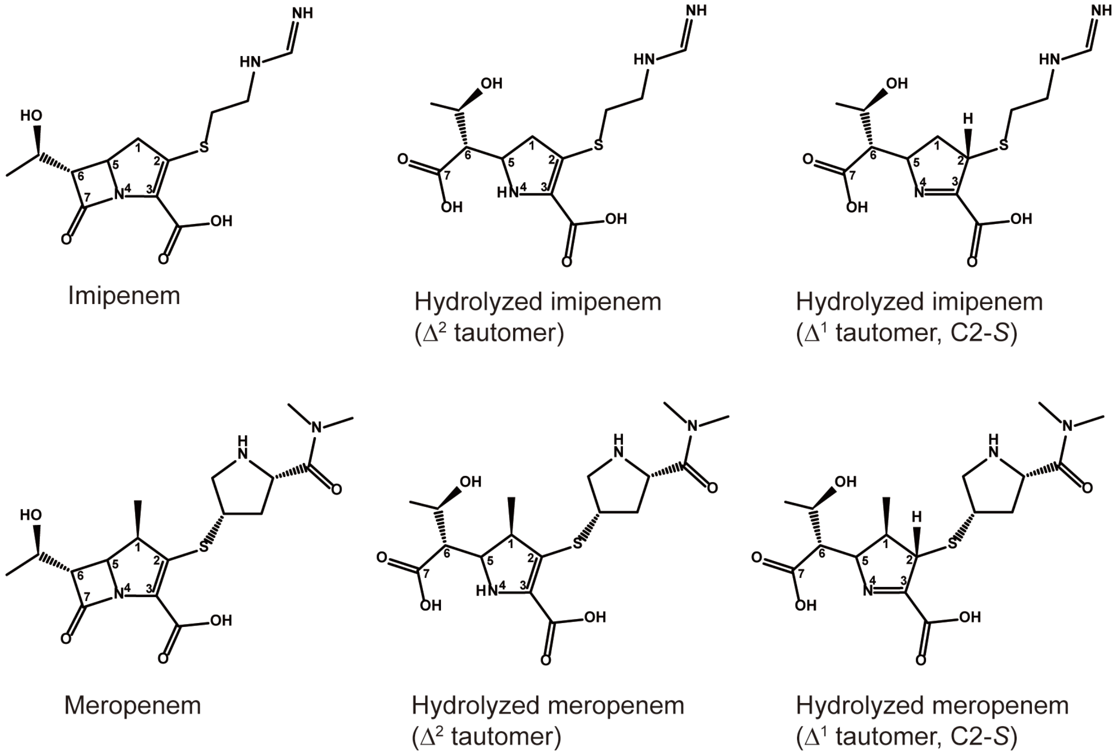

Supplementary Figure 1. Chemical structures of imipenem and meropenem that were used as β-lactam antibiotic substrates in this study. The Δ^2 tautomer of hydrolyzed antibiotic molecules was modeled in the crystal structures representing the EI_1 intermediates, while the Δ^1 tautomer (*S* chirality at position 2) was modeled in the structures representing the EI_2 intermediates and the EP complex.

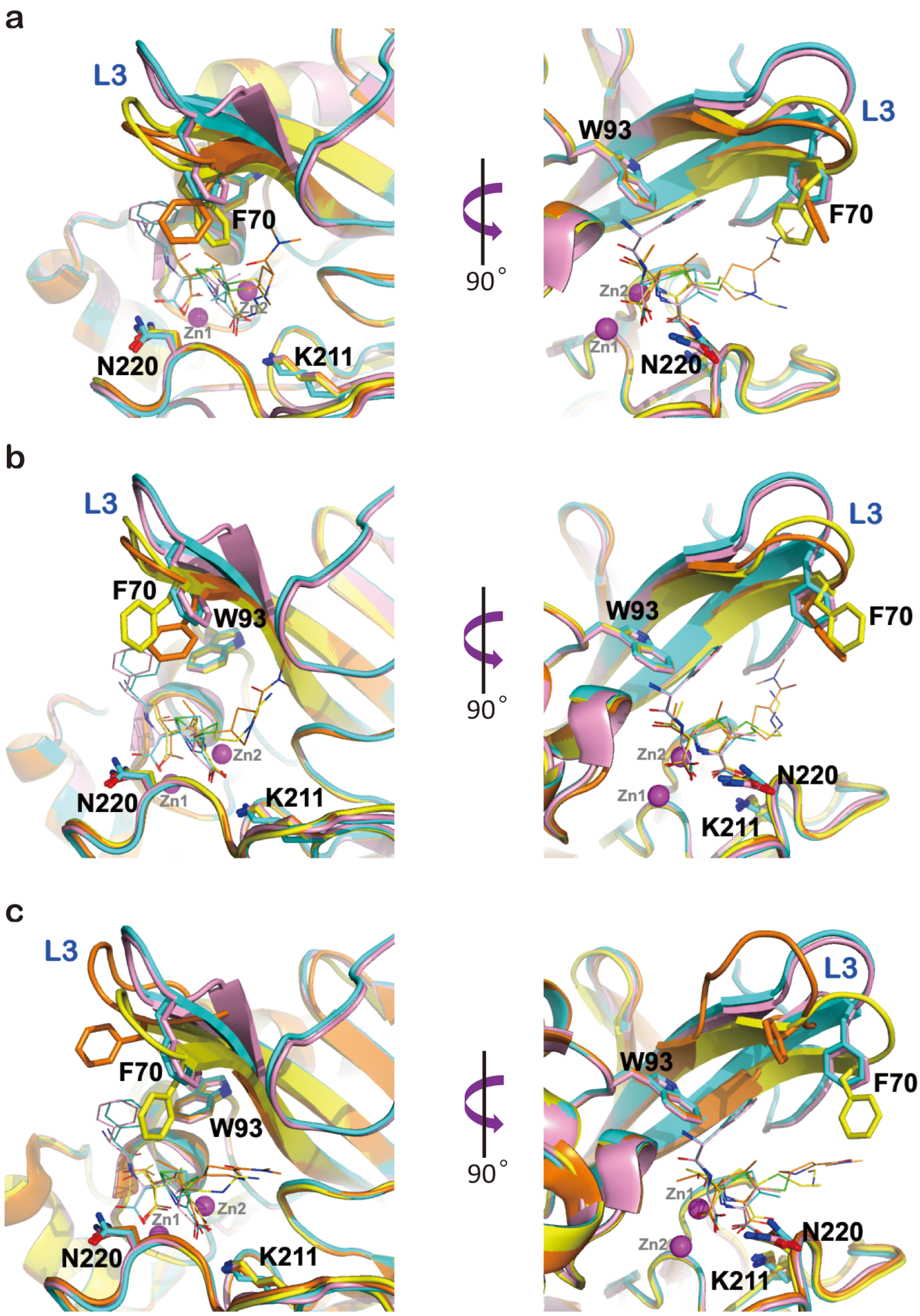

Supplementary Figure 2 Superimposition of the active site in NDM-1 bound with imipenem, meropenem, ampicillin and cephalexin to compare the orientations and positions of F70, W93, K211 and N220. Imipenem (yellow) and meropenem (orange) bound structures representing the EI_1 (**a**) or EI_2 (**b**) intermediates, and the EP complex of NDM-1/imipenem solved in our study (yellow) and the structure of PDB 4EYL[1] (orange) (**c**) are overlaid with ampicillin (pink) (PDB 3Q6X)[2] and cephalexin (cyan) (PDB 4RL2)[3] bound structures. The hydrolyzed antibiotics are represented in line models and the zinc ions are denoted by magenta spheres.

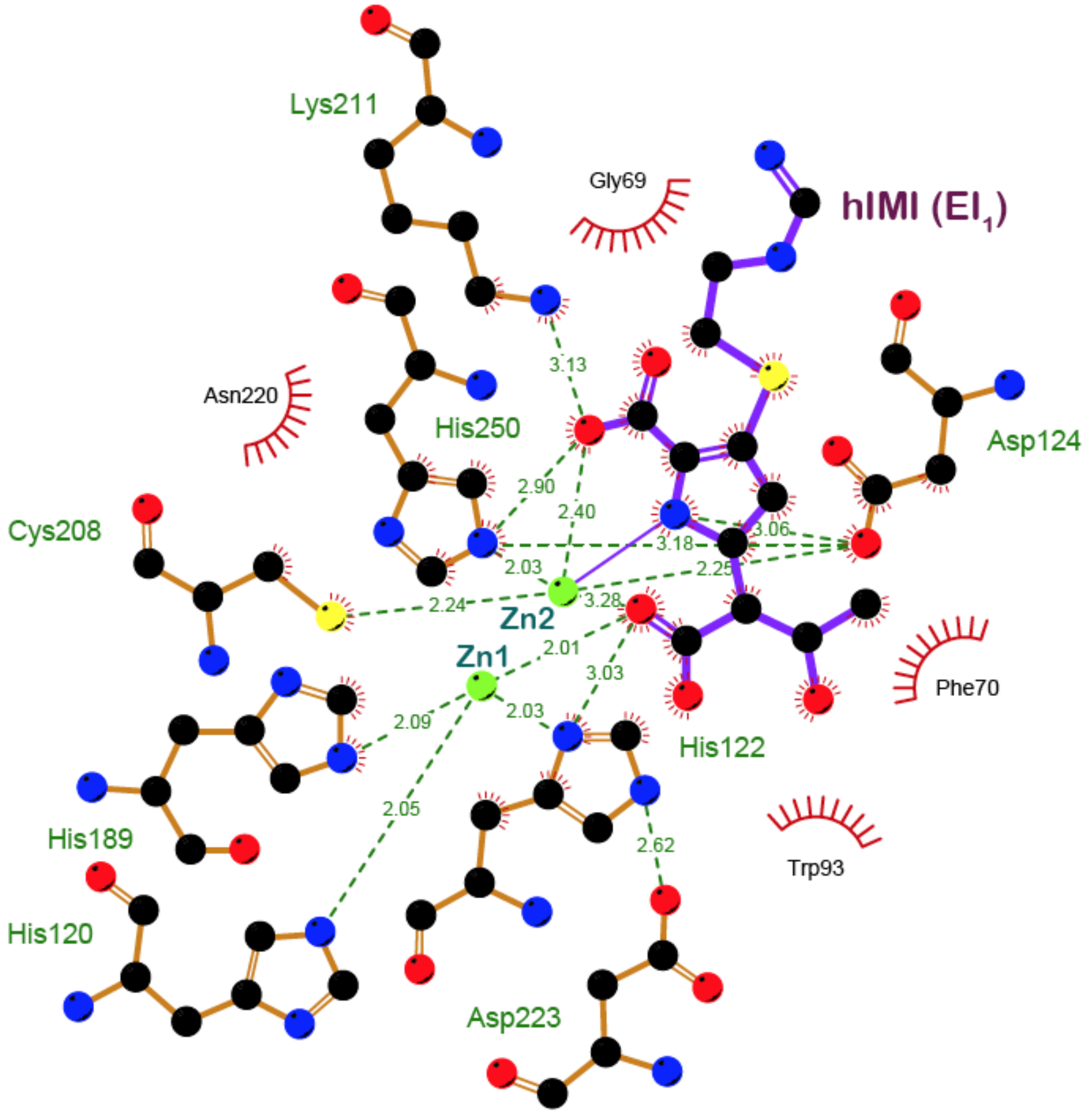

Supplementary Figure 3. Ligand-protein and ligand-Zn^{2+} interactions around hydrolyzed imipenem that was modeled in the structures representing the EI_1 complex (Fig. 2a) schematically depicted using LigPlot[4]. Hydrogen bonds are shown as dashed lines and the amino acids involved in forming hydrophobic interactions with the bound β-lactam antibiotics are displayed as indented curves. The labeled numbers are the hydrogen bond lengths in Å.

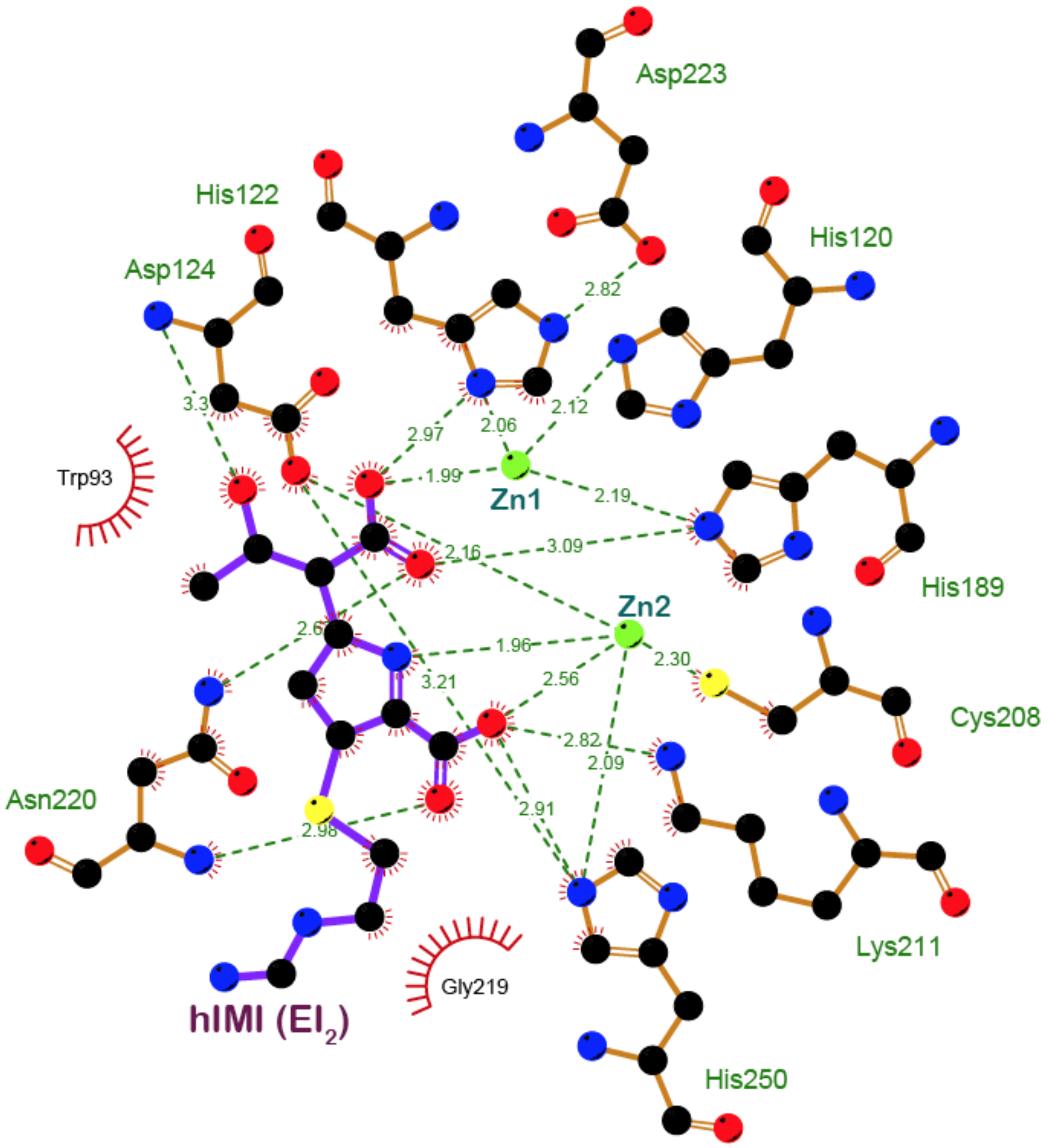

Supplementary Figure 4. Ligand-protein and ligand-Zn^{2+} interactions around hydrolyzed imipenem that was modeled in the structures representing the EI_2 complex (Fig. 2b and Fig. 3b) schematically depicted using LigPlot[4]. Hydrogen bonds are shown as dashed lines and the amino acids involved in forming hydrophobic interactions with the bound β-lactam antibiotics are displayed as indented curves. The labeled numbers are the hydrogen bond lengths in Å.

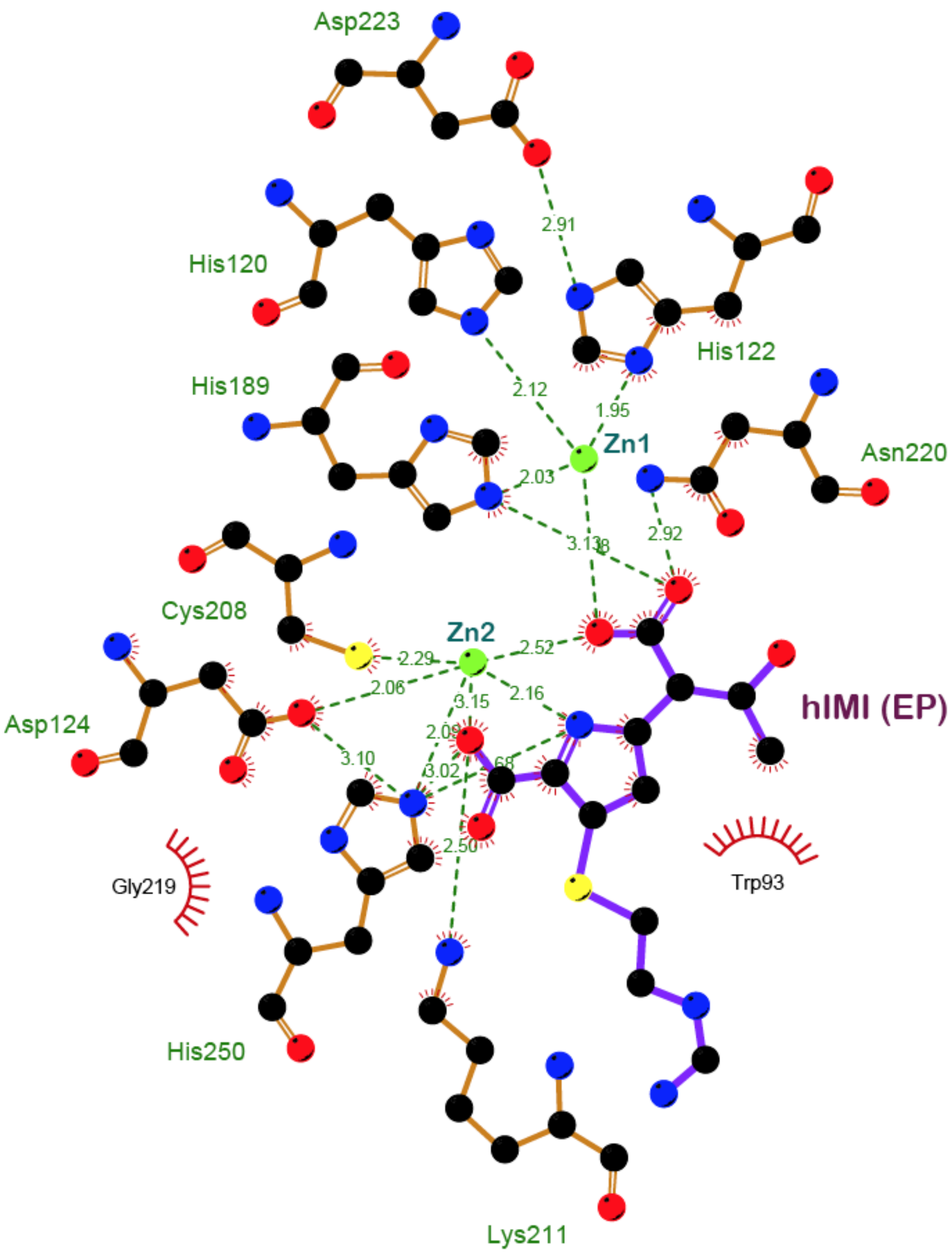

Supplementary Figure 5. Ligand-protein and ligand-Zn^{2+} interactions around hydrolyzed imipenem that was modeled in the structures representing the EP complex (Fig. 2c and Fig. 3c) schematically depicted using LigPlot[4]. Hydrogen bonds are shown as dashed lines and the amino acids involved in forming hydrophobic interactions with the bound β-lactam antibiotics are displayed as indented curves. The labeled numbers are the hydrogen bond lengths in Å.

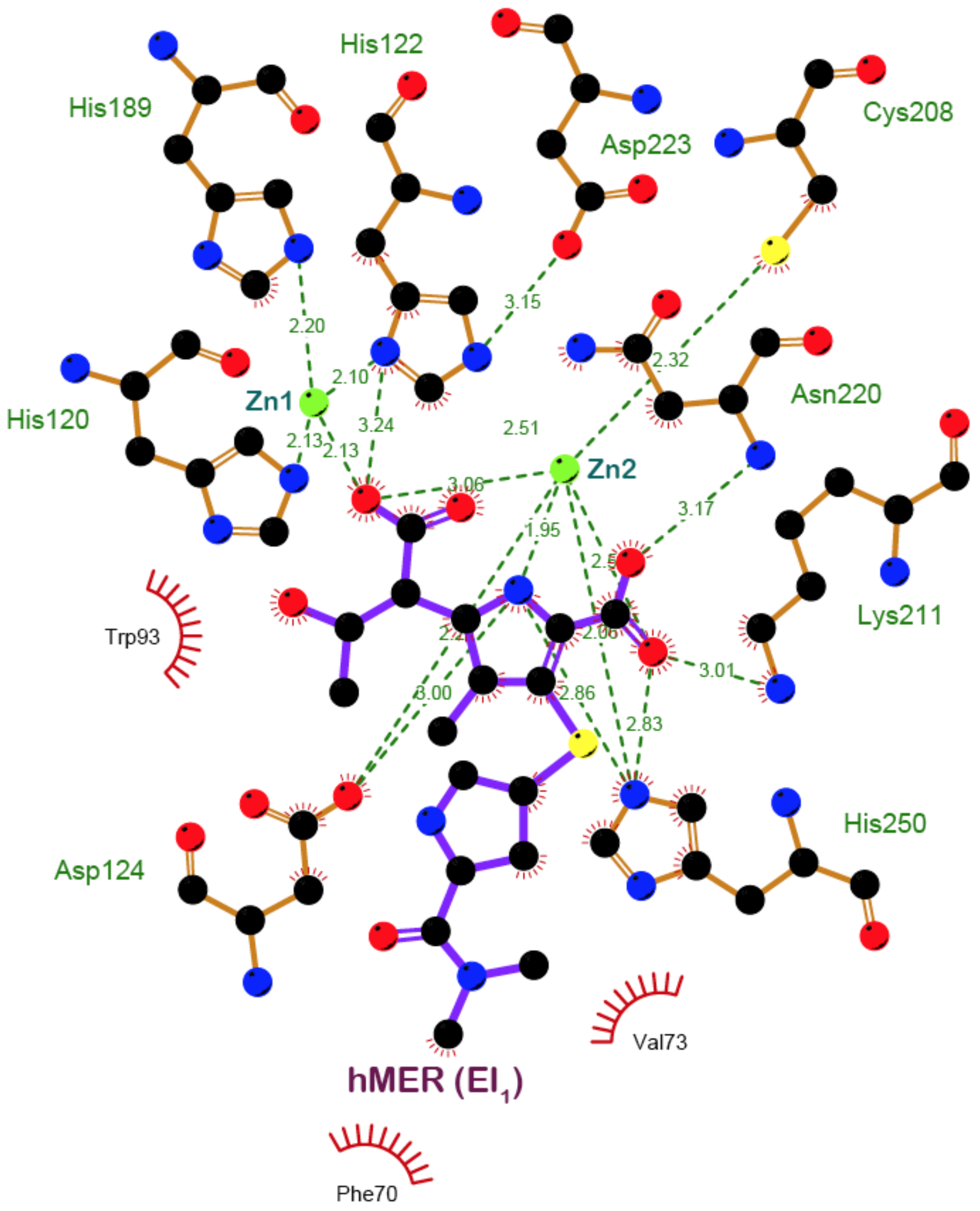

Supplementary Figure 6. Ligand-protein and ligand-Zn^{2+} interactions around hydrolyzed meropenem that was modeled in the structures representing the EI_1 complex (Fig. 2d and Fig. 3a) schematically depicted using LigPlot[4]. Hydrogen bonds are shown as dashed lines and the amino acids involved in forming hydrophobic interactions with the bound β-lactam antibiotics are displayed as indented curves. The labeled numbers are the hydrogen bond lengths in Å.

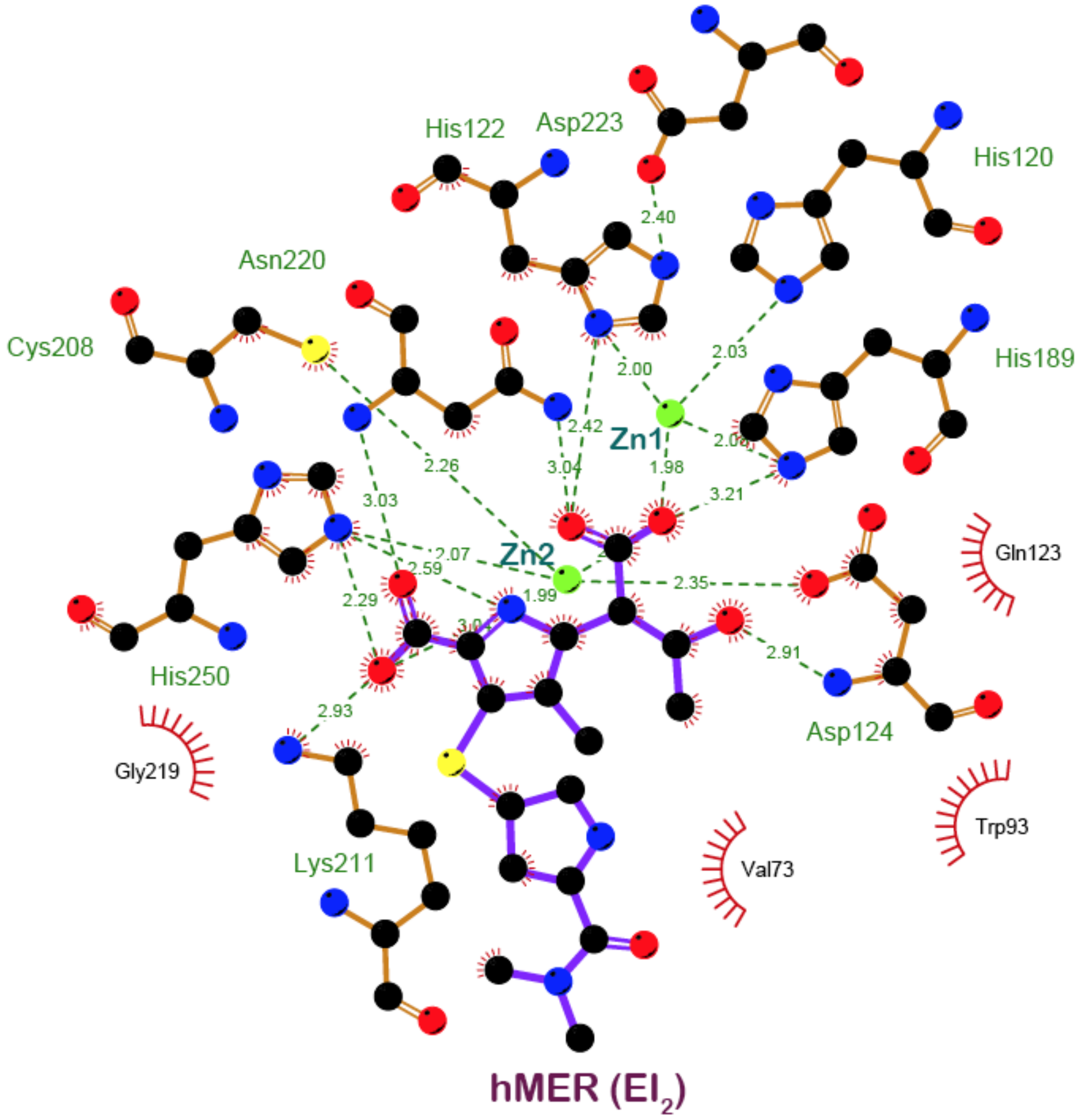

Supplementary Figure 7. Ligand-protein and ligand-Zn^{2+} interactions around hydrolyzed meropenem that was modeled in the structures representing the EI_2 complex (Fig. 2e) schematically depicted using LigPlot[4]. Hydrogen bonds are shown as dashed lines and the amino acids involved in forming hydrophobic interactions with the bound β-lactam antibiotics are displayed as indented curves. The labeled numbers are the hydrogen bond lengths in Å.

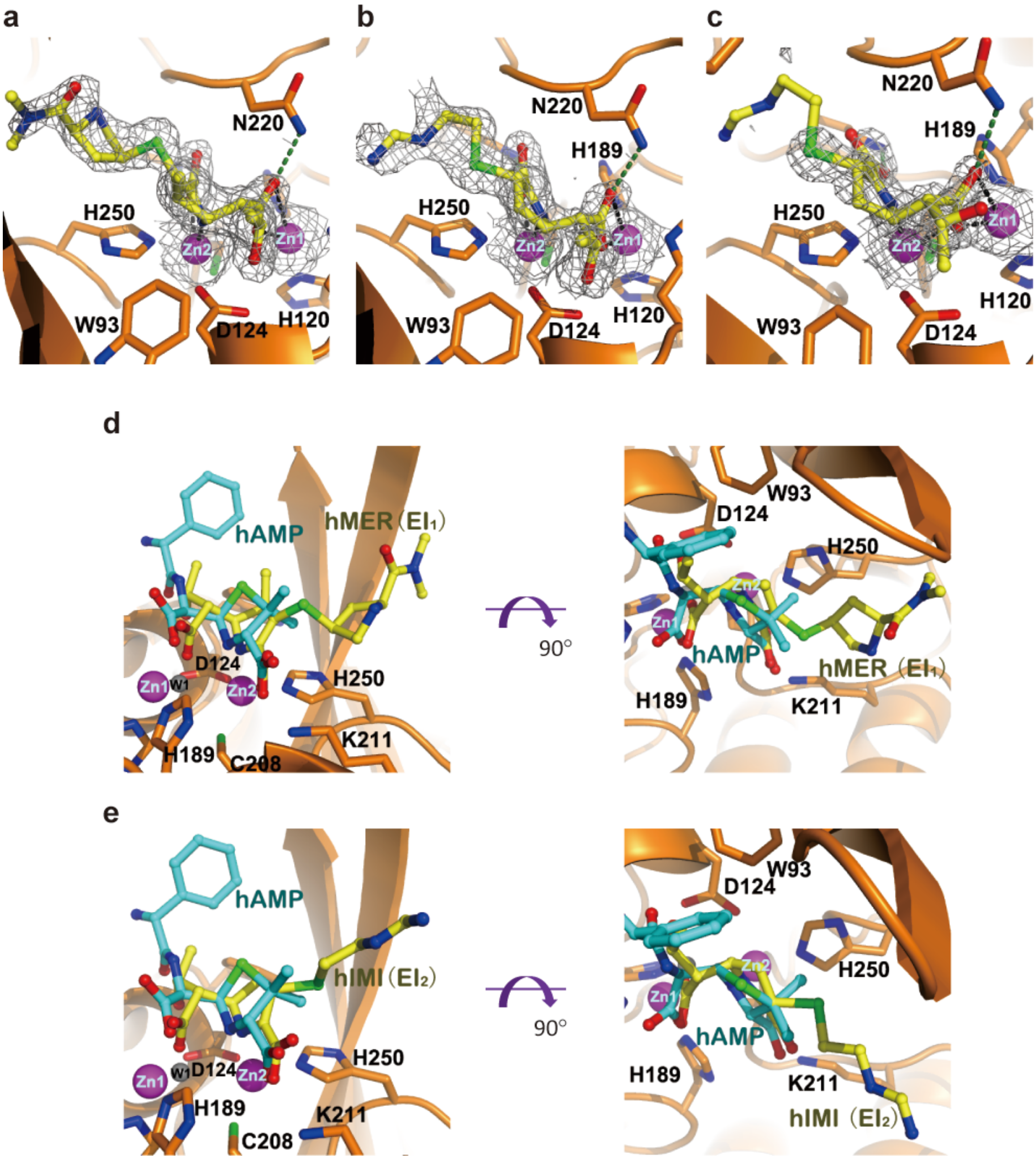

Supplementary Figure 8. Close-up views of the active site of NDM-1 representing different enzyme-intermediate/product adducts from a viewing angle different from Figure 3. (**a-c**), Representation of hydrolytic intermediates of mereopenem (hMER) present in EI_1 (**a**) and imipenem (hIMI) present in EI_2 (**b**) or EP (**c**) viewing from an angle by rotating 180° along the *y* axis with regard to the same representations shown in panel **a-c** of Figure 3. (**d-e**), Structure comparison of hydrolyzed amipicillin (hAMP) (PDB 3Q6X)[2] with hMER present in EI_1 (**d**) or hIMI present in EI_2 (**e**).

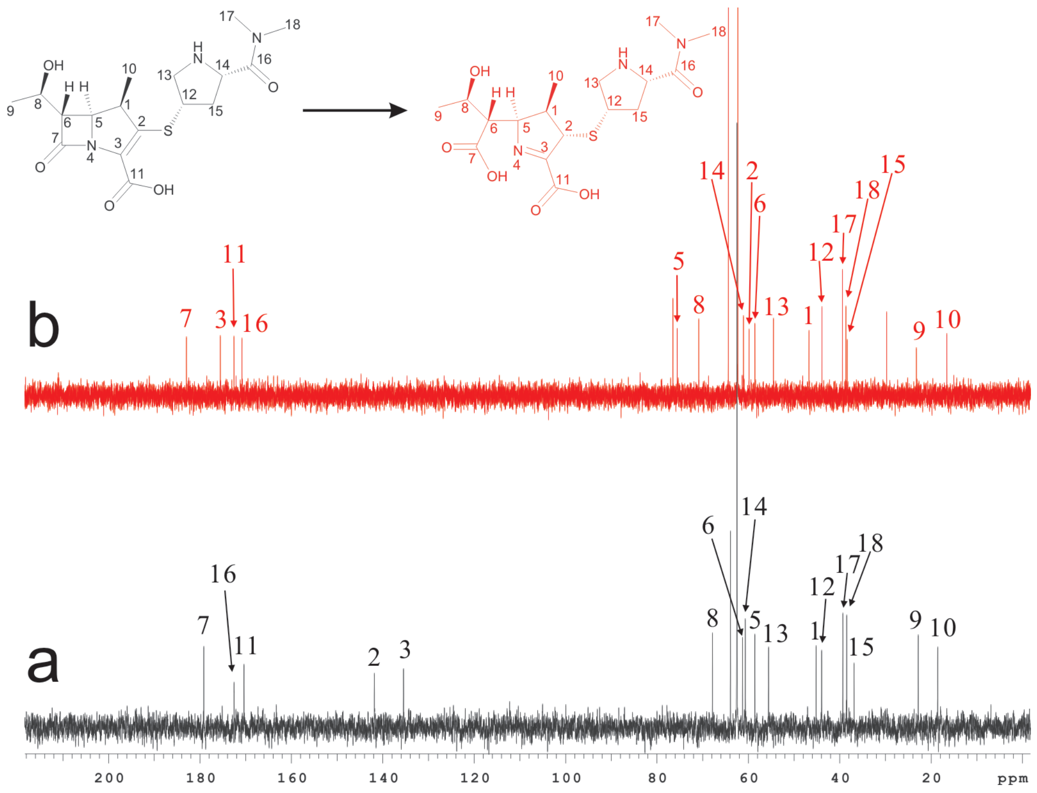

Supplementary Figure 9. ^{13}C NMR spectra of meropenem hydrolysis catalyzed by NDM-1 in Tris-HCl buffer. The spectra recorded for the intact substrate (**a**) and the final hydrolyzed product (**b**) are shown in parallel to compare the chemical shifts before and after the hydrolysis. The chemical structures of the substrate and the product are displayed on upper-left in black and red respectively.

Supplementary Table 1. Synthesized nucleotide sequence encoding NDM-1 and primer sequences used in PCR amplification

**Synthesized nucleotide sequence**	5’-ATGGAATTGCCCAATATTATGCACCCGGTCGCGAAGCTGAGCACCG<br>CATTAGCCGCTGCATTGATGCTGAGCGGGTGCATGCCCGGTGAAATCC<br>GCCCGACGATTGGCCAGCAAATGGAAACTGGCGACCAACGGTTTGGCG<br>ATCTGGTTTTCCGCCAGCTCGCACCGAATGTCTGGCAGCACACTTCCT<br>ATCTCGACATGCCGGGTTTCGGGGCAGTCGCTTCCAACGGTTTGATCG<br>TCAGGGATGGCGGCCGCGTGCTGGTGGTCGATACCGCCTGGACCGATG<br>ACCAGACCGCCCAGATCCTCAACTGGATCAAGCAGGAGATCAACCTGC<br>CGGTCGCGCTGGCGGTGGTGACTCACGCGCATCAGGACAAGATGGGCG<br>GTATGGACGCGCTGCATGCGGCGGGGATTGCGACTTATGCCAATGCGT<br>TGTCGAACCAGCTTGCCCCGCAAGAGGGGATGGTTGCGGCGCAACACA<br>GCCTGACTTTCGCCGCCAATGGCTGGGTCGAACCAGCAACCGCGCCCA<br>ACTTTGGCCCGCTCAAGGTATTTTACCCCGGCCCCGGCCACACCAGTG<br>ACAATATCACCGTTGGGATCGACGGCACCGACATCGCTTTTGGTGGCT<br>GCCTGATCAAGGACAGCAAGGCCAAGTCGCTCGGCAATCTCGGTGATG<br>CCGACACTGAGCACTACGCCGCGTCAGCGCGCGCGTTTGGTGCGGCGT<br>TCCCCAAGGCCAGCATGATCGTGATGAGCCATTCCGCCCCCGATAGCC<br>GCGCCGCAATCACTCATACGGCCCGCATGGCCGACAAGCTGCGCTGA-<br>3′
**Forward primer**[1]	5′-ATATTAA<u>CATATG</u>GGTGAAATCCGCCCG-3′
**Reverse primer**[2]	5′- TTA<u>CTCGAG</u>TCAGCGCAGCTTGTCGGCC-3′

[1] Underlined is the *Nde*I restriction site.

[2] Underlined is the *Xho*I restriction site.

Supplementary Table 2. Chemical shifts of meropenem before and after hydrolysis in ^{1}H and ^{13}C spectra

Atom index	Meropenem				Hydrolyzed meropenem			
	^{1}H (ppm)	Integ.	Multi.	^{13}C (ppm)	^{1}H (ppm)	Integ.	Multi.	^{13}C (ppm)
1	3.35	1	m	45.14	2.51	N/A	m	46.43
2	NA	N/A	N/A	140.67	3.87	N/A	s	59.74
3	NA	N/A	N/A	136.09	NA	N/A	N/A	176.17
5	4.20	ov	ov	58.61	4.31	N/A	dd	75.01
6	3.42	ov	ov	61.43	2.62	N/A	dd	57.89
7	NA	N/A	N/A	179.25	NA	N/A	N/A	182.22
8	4.21	ov	ov	67.84	4.00	N/A	dt	70.44
9	1.25	3	d	22.85	1.22	N/A	d	22.92
10	1.17	3	d	18.57	1.03	N/A	d	16.32
11	NA	N/A	N/A	170.30	NA	N/A	N/A	171.99
12	4.01	1	m	43.14	3.76	N/A	ov	43.92
13	3.73, 3.42	1, ov	dd, ov	55.03	3.77, 3.39	N/A	ov, dd	54.39
14	Water signal*	N/A	N/A	60.89	Water signal*	N/A	N/A	60.98
15	3.14, 1.92	ov, 1	ov, m	36.36	2.98, 1.89	N/A	ov, m	38.31
16	NA	N/A	N/A	170.74	NA	N/A	N/A	170.70
17, 18	3.02, 2.96	3, 3	s, s	39.32, 38.56	3.02, 2.95	N/A	s, s	39.35, 38.61

* Proton signal merged with water signal.

Supplementary References:

1 King, D. T., Worrall, L. J., Gruninger, R. & Strynadka, N. C. New Delhi metallo-beta-lactamase: structural insights into beta-lactam recognition and inhibition. *J Am Chem Soc* **134**, 11362-11365 (2012).

2 Zhang, H. & Hao, Q. Crystal structure of NDM-1 reveals a common beta-lactam hydrolysis mechanism. *FASEB J* **25**, 2574-2582 (2011).

3 Feng, H. *et al.* Structural and mechanistic insights into NDM-1 catalyzed hydrolysis of cephalosporins. *J Am Chem Soc* **136**, 14694-14697 (2014).

4 Laskowski, R. A. & Swindells, M. B. LigPlot+: multiple ligand-protein interaction diagrams for drug discovery. *J Chem Inf Model* **51**, 2778-2786 (2011).
